# PALTEM: What Parameters Should Be Collected in Disaster Settings to Assess the Long-Term Outcomes of Famine?

**DOI:** 10.3390/ijerph15050857

**Published:** 2018-04-25

**Authors:** Alexandra Moraru, Maria Moitinho de Almeida, Jean-Marie Degryse

**Affiliations:** 1 Centre for Research on the Epidemiology of Disasters, Université Catholique de Louvain, Brussels 1200, Belgium; maria.rodrigues@uclouvain.be; 2 Institute of Health and Society, Université Catholique de Louvain, Brussels 1200, Belgium; jean-marie.degryse@uclouvain.be; 3 Department of Public Health and Primary Care, Katholieke Universiteit Leuven, Leuven 3000, Belgium

**Keywords:** environmental epigenetics, DNA methylation, life-stage exposure, famine, risk assessment

## Abstract

Evidence suggests that nutritional status during fetal development and early life leaves an imprint on the genome, which leads to health outcomes not only on a person as an adult but also on his offspring. The purpose of this study is to bring forth an overview of the relevant parameters that need to be collected to assess the long-term and transgenerational health outcomes of famine. A literature search was conducted for the most pertinent articles on the epigenetic effects of famine. The results were compiled, synthesized and discussed with an expert in genetics for critical input and validation. Prenatal and early life exposure to famine was associated with metabolic, cardiovascular, respiratory, reproductive, neuropsychiatric and oncologic diseases. We propose a set of parameters to be collected in disaster settings to assess the long-term outcomes of famine: PALTEM (parameters to assess long-term effects of malnutrition).

## 1. Introduction

Famines affect millions of people worldwide every year, causing psychological and physiological stress to the human body, with a potential risk to affect the unborn child. According to the Food Security Information Network, there are four countries at risk of famine: South Sudan, Somalia, Yemen and northeast Nigeria, where more than 20 million people are affected by starvation [1]. This is the biggest humanitarian crisis since 1945, and it is yet unknown how it will affect the health of consecutive generations. Until now, only the short-term impact of famine has been evaluated, but the consequences of the emerging food crisis represent a global and lasting public health burden, with long-term impacts not only on mental health but also on the overall physical state. Several research studies, however, have investigated the long-term consequences of famine. To our knowledge, there have been no efforts to examine existing research and summarize the health parameters that can predict the long-term impact of famine.

We hypothesize that a way to better predict and prevent these consequences is collecting information at a blind phase and making correlations between epigenetic markers and health parameters. The aim of this study, therefore, is to determine the long-term effects of fetal and infant exposure to famine and to bring forth an exhaustive summary of relevant parameters to predict them. We expect the results of this work to be a first step in aiding field health workers in collecting relevant information on the health outcomes of famine exposure.

### 1.1. Epigenetic Mechanisms

**Figure 1:**
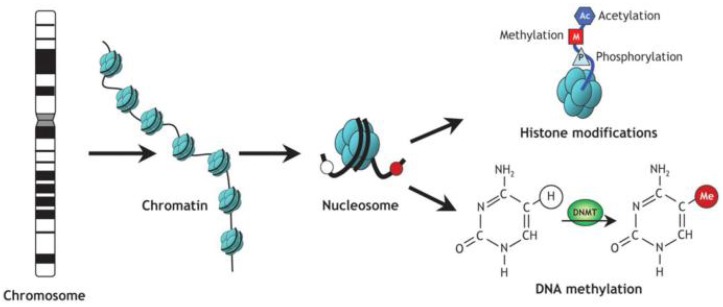
Schematic of epigenetic modifications. Strands of DNA are wrapped around histone octamers, forming nucleosomes, which organize the chromatin. Reversible and site-specific histone modifications occur at multiple sites through acetylation, methylation and phosphorylation. DNA methylation occurs at the 5-position of cytosine residues in a reaction catalyzed by DNA methyltransferases (DNMTs). Together, these modifications provide a unique epigenetic signature that regulates chromatin organization and gene expression [6].

Epigenetic studies have assessed deoxyribonucleic acid (DNA) methylation and histone modifications (Figure 1). These processes, rather than altering the DNA sequence, interfere in the expression of genes, causing tissue-specific changes. The DNA methylation process involves two functions: suppressing gene expression and maintaining genome integrity by suppressing repetitive elements and heterochromatin [2]. Methylation changes the chromatin structure and affects transcription as a result of the interaction between DNA and proteins [3]. In some circumstances, methylation interferes directly with transcription binding, preventing the latter [4]. DNA methyltransferases (DNMTs) are enzymes that maintain the methylation signals and execute a de novo methylation. Gene expression is regulated post-transcriptionally by a short non-coding ribonucleic acid, called a microRNA (miRNA). It controls the expression of epigenetic regulators, such as DNMTs and histone deacetylases. Epigenetics regulate the gene expression pattern transcriptionally and post-transcriptionally, controlling and forming a regulatory circuit and maintaining normal physiological functions [5].

### 1.2. Epigenetics and Environmental Exposure to Famine

The Fetal Origin of Health and Disease (FOHaD), or Barker’s Hypothesis, was formulated in 1995 and suggested that the environment was an important factor for growth and early development and could be a risk factor for cardiovascular disease (CVD) [7]. This was based on findings in Hertfordshire, which showed a positive correlation between lower birth weight and death rate due to ischemic heart disease [8].

The FOHaD theory advanced to the Developmental Origin of Health and Disease (DOHaD). It postulates that in addition to the environmental factors that affect the fetus in utero, several other factors occurring in the early years of development also imprint health outcomes for a person and its offspring. The DOHaD offers realistic, accurate, and integrative approaches to understanding the environmental disruption of developmental programming [9].

According to the DOHaD, early life exposure to famine is a key factor of the causes of diseases. Cohort studies were set up to investigate the influence of prolonged fasting on the current health status. There is evidence for the epigenetic effects of famine on the population’s health [10].

**Figure 2:**
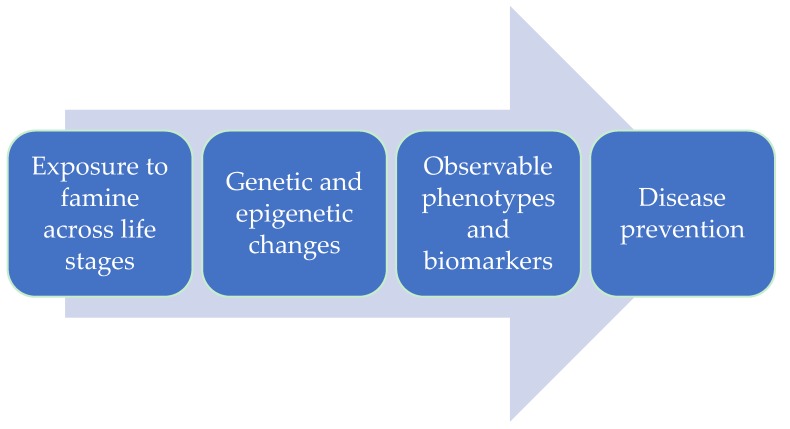
Translating epigenetic markers of famine exposure to public health interventions. (*a*) Environmental exposures throughout life induce epigenetic and genetic alterations, particularly in susceptible populations. (*b*) Epigenetic and genetic changes serve as molecular biosensors of environmental exposure effects, and these effects can be quantified within populations. (*c*) Epigenetic and genetic changes could presage observable phenotypes, including both disease phenotypes and biomarkers, indicative of disease. (*d*) At-risk individuals and subpopulations identified by molecular sensors and biomarkers can be targeted for public health intervention [11].

This review is focused on finding the transgenerational effects of famine, based on previous studies on exposure to caloric restriction to find correlations between epigenetic markers and parameters that can be assessed in disaster settings (Figure 2). We aimed to bring forth a set of parameters to be collected at an early stage of exposure, enabling the assessment of the transgenerational effects of famine many years later.

## 2. Materials and Methods

### 2.1. Identifying the Research Question

In this study, we focused on the health effects of famine as changes in body function or cell structure that led to disease or health problems [12]. A scoping review was performed according to the methodology derived from Arksey and O’Malley’s paper [13] by doing a back-and-forth search between early findings and new insights and changing the search terms during the process.

### 2.2. Finding Relevant Studies

An initial search of the MEDLINE (PUBMED) database was performed by AM to identify articles on the Dutch Famine Cohort Study. Because the focus of the study was to explore the question widely, the chosen database served as a relevant starting point to study the matter broadly. The reference lists were then scanned to find other relevant articles. Five other cohorts were identified and selected, where the transgenerational effects of famine had been investigated (Table 1). A description of the cohorts is found in Appendix A.

### 2.3. Selecting the Studies

**Figure 3:**
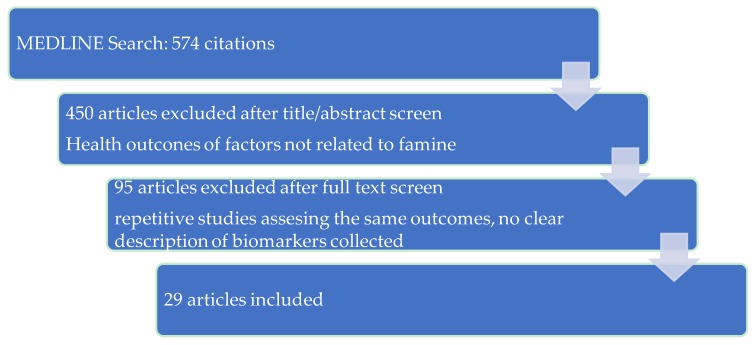
Selection process of eligible studies.

After selecting these relevant cohorts, an extensive search in the MEDLINE (PUBMED) database for articles related to them was performed and AM and JMD selected the articles according to the eligibility criteria. Inclusion criteria for this review were as follows: published English-language research on humans that reported health effects from famine. Articles were included if they assessed at least one health outcome of famine. Articles lacking a good representation of the study methods and assessed parameters were excluded. The initial search produced 574 publications. Each retrieved article was assessed by a thorough examination of its abstract and, if needed, its full content. References were searched, reviewed and documented by AM, JMD and MMA to avoid any omissions. After assessing eligibility, 29 articles were included in this study (Figure 3).

### 2.4. Charting the Information

All results were exported to an EndNote library. The full text of the selected articles was read, and an information synthesis on the transgenerational effects of famine was compiled in a table (Appendix B).

### 2.5. Summarizing the Results

Based on these findings, a list of parameters were identified, compiled and ordered according to the financial resources needed for implementation. This list was then presented to an expert in human genetics who provided critical inputs and validated a final set of parameters.

## 3. Results

### 3.1. Common Health Outcomes

The most common health outcomes found among all six cohorts are higher body mass index (BMI) from infancy till late adulthood, elevated risk for obesity, type 2 diabetes mellitus (T2DM), disturbances on the diencephalic–hypothalamic–pituitary axis (DHPA) with an impact on hormone secretion [14], microalbuminuria, proteinuria, hypertension, ischemic heart disease (IHD) and cerebrovascular disease (CBD) (Table 2).

### 3.2. Metabolic Outcomes

De Rooij et al. reported elevated levels of triacylglycerol concentrations among exposed subjects and suggested that fetal undernutrition influences the elements of metabolism [15]. Increased BMI among the grandchildren of men exposed to famine in utero was reported [10]. One hypothesis is the change in dietary preferences, with a tendency to consume high-fat diet among subjects exposed to famine in early gestation, and a more atherogenic lipid profile later in life [16]. Strong correlation between famine exposure in utero or in infancy and metabolic syndromes was found among the Chinese cohort. The risk among subjects who followed a Western diet was exacerbated [17]. Wang et al. found an increased prevalence of T2DM because of famine exposure in utero or in infancy followed by high economic status in adulthood [18].

Insulin resistance is a common pathogenic mechanism for hyperglycemia and hypertension [19]. The effects of intrauterine famine exposure on glucose metabolism is explained by reduced skeletal muscle development, which leads to insulin resistance in peripheral tissues [20]. Roseboom et al. concluded that the impaired glucose tolerance (IGT) among those exposed to famine in early and mid-gestation is mediated through an insulin secretion defect [21].

Exposure to famine in infancy showed increased rates in developing osteoporosis in adulthood [22], often with premature onset [23].

### 3.3. Cardiovascular Disease

An increased mortality among men, but not women, due to IHD and CBD was revealed among the survivors of the siege of Leningrad [24,25]. Famine during puberty showed the strongest correlations with systolic blood pressure (SBP) and stroke, compared to exposure at other ages [24]. The Dutch Famine Birth Cohort Study revealed that people exposed to famine in utero showed a higher incidence of coronary artery disease (CAD), which occurred at an earlier age [26].

Nigerian and Chinese populations exposed to famine in utero or in infancy showed increased blood pressure (BP) and body weight [27,28].

### 3.4. Respiratory Disease

People who experienced famine during infancy and adolescence had an increased risk for hospitalization due to asthma, chronic obstructive pulmonary disease (COPD), and obstructive airways disease (OAD) [29]. Among those exposed to famine in mid-gestation, the prevalence of OAD was increased, with higher rates among those exposed in early gestation [30]. These outcomes were associated with reduced lung function or Immunoglobulin E (IgE) concentration, suggesting that an increased bronchial reactivity, rather than irreversible airflow obstruction, could have caused it [21].

### 3.5. Reproductive Outcomes

Women from China exposed to famine in utero had an increased risk for permanent impaired fecundity [31]. Menstrual irregularities and a longer period from menarche to regular menses were found among Dutch women exposed to famine in infancy [32] and earlier menopause among those exposed to famine in utero [33].

### 3.6. Neuropsychiatric Outcomes

De Rooj SR et al. found that fetal undernutrition permanently affects brain size. Prenatally exposed men, but not women, showed a smaller brain and intracranial volume [34]. Among prenatally exposed men, congenital abnormalities of the central nervous system, schizophrenia and schizophrenia spectrum personality disorders (SSPD) were found [35]. Prenatal exposure to severe undernutrition also has negative effects on visual-motor skills, mental flexibility and selective attention in adulthood [36].

### 3.7. Oncologic Outcomes

Infancy exposure to the Chinese famine was associated with elevated risk for esophageal, gastric, liver and colorectal cancer, with unknown underlying mechanisms [37]. An overall increased risk for malignancy, especially breast and colorectal cancer, was observed among Holocaust survivors [38].

A study among Dutch women exposed to famine showed an increase in postmenopausal hormones and breast cancer risks [14]. These findings are explained by the raised levels of growth factors, which is a result of fetal growth restriction, followed by rapid postnatal growth, which can cause the growth of pre-malignant cells [39]. Subjects exposed to famine postnatally had an increased risk for breast cancer, and the age between 2 and 10 years was found to be a susceptible window for breast malignancy [14,40].

### 3.8. Others

Chen et al. found an association between the exposure in utero or in infancy to Chinese famine and an increased risk for fatty liver disease in adulthood [41]. Increased levels of fibrinogen concentrations and lower levels of factor VII concentrations were found among the Dutch Famine Cohort, suggesting that famine exposure in utero affects liver function [42].

High levels of protein in urine were found among subjects exposed to Chinese famine in utero or in infancy [43]. Among those exposed to Dutch famine in mid-gestation, renal function was affected in adulthood, with a 3.2-fold increase in occurrence of microalbuminuria and a 10% decrease in creatinine clearance [44]. The authors assume that gestation has organ-specific periods that are sensitive to environmental factors and can have health outcomes later in life.

### 3.9. Proposed Parameters

Proxy indicators of food restriction severity before birth include maternal pre-pregnancy BMI (ppBMI), gestational weight gain (GWG), mother’s glucose level, and child’s weight and size at birth. Higher maternal ppBMI, GWG and glucose level were associated with higher birth weight and adiposity in early infancy [45]. Overweight infants had a four-fold risk for obesity when adults and several co-morbidities throughout the life course [46]. An early detection of these risks is a key factor in preventing obesity occurrence. It is a feasible method in disaster settings, due to low resources and the untrained health personnel needed.

Clinical measurements can be used to predict the risk for metabolic diseases, such as obesity, impaired glucose tolerance (IGT), T2DM and CAD. Thicker fat tissue is another marker of later obesity and T2DM. Faster heart rate (HR) is associated with lower DNA methylation at two adjacent cytosine phosphate guanine (CpG) dinucleotides and represents a risk factor for coronary heart disease (CHD) and increased cardiovascular mortality [47]. Higher pulse wave velocity (PWV) is a marker for elevated arterial stiffness and leads to greater cardiovascular risk [47]. These measurements are easy to perform by healthcare workers with minimal resources.

Along with discoveries in genetic and epigenetic fields, the risk for metabolic diseases, CVD, cancer and some mental disorders can be assessed by DNA methylation tests. To perform an epigenomic profiling, blood samples must be collected, processed and stored at specific conditions. It was proven that DNA methylation in blood is similar to changes in pancreatic islets and can be used as a biomarker of insulin secretion and T2DM [48]. There are epigenetic markers used for early detection of CVD [49]. Osteoarthritis (OA) and cellular senescence are driven by some epigenetic alterations, according to McCulloch et al. The currently reported data are of great relevance for biobanking and further research to better understand disease mechanisms.

Previous studies demonstrated the relationship between fetal and infant exposure to famine and the risk for cancer. DNA methylation can begin very early in the breast tumor [50], creating favorable conditions for the early-stage detection of malignancy [51]. Epigenetic alterations and markers were investigated to depict the risk for gastric cancer [52]. Colorectal cancer can be diagnosed by DNA methylation and gene expression analysis. These markers can be used for early detection and prognosis for malignancy [53]. Qualified and trained personnel, sterile single-use equipment and expensive storing conditions are needed to perform DNA methylation tests. Alternative methods were studied and accepted among researchers.

Staunstrup et al. demonstrated high levels of comparability between the DNA from archived dried blood samples (DBS) and from freshly prepared DBS [54]. This method requires low amounts of blood and filter cards for storing the samples at room temperature for an indefinite period. It can be used for intervention measures and further epigenetic research.

Investigations on human saliva are increasingly popular because of its accessibility, availability and non-invasive applicability. This method provides time-sensitive information at low expenses, complemented with innovations with respect to the microbiome, proteome, transcriptome and epigenome [55].

Using the proposed parameters, together with findings on the epigenetic effects of famine, correlations can be made, and health outcomes can be predicted at the prevention level. A list of parameters is hereby proposed for field application to aid public health workers in preventing the diseases that occur late in life as a transgenerational effect of famine (Table 3).

## 4. Discussion

Efforts have been made to systematically summarize the outcomes of prenatal exposure to famine. However, this paper is the first one, to our knowledge, to bring forth a set of parameters that can be transposed to field actions in order to gather data at an early stage and assess these outcomes many years later.

Epigenetics is a novel field of science, and there is still converging evidence of epigenetic effects. There are correlations with epigenetic implications, but further studies are needed to distinguish clearly the epigenetic effects from the genetic ones. What researchers revealed as consequences of famine are a combination of genetic effects and the additional effects of famine. This explains how consequences of the same factor, such as the daily amounts of calories received during a specific period, differ from individual to individual. A combination of genome and epigenome studies must be performed to obtain a complete picture of the consequences of famine.

Assessing the retrospective and current health status using surveys is a cost-effective method. Healthcare workers with a background in data collection are required. Retrospective data on the birth size and weight, mother’s ppBMI, GWG and glucose level, supplemented with data on actual dietary habits, can offer important information on the risk for cardiovascular and metabolic diseases in childhood and later in life. Obesity, IGT, T2DM, CAD and CVD are just a few of the health outcomes but are most common among famine survivors. The value of this tool is to be considered, especially when it may be the only one available.

Another relatively low-cost parameter is clinical measurement, namely, skinfold thickness, hip:waist ratio and BMI. These offer information on current health status and the risk for metabolic diseases. Complemented with HR and PWV measurements, the data can be used to assess the risk for CVD.

To understand the long-term effects of nutrient deprivation on health implies genetic and epigenetic analysis of biological tissue. The most accepted material among researchers and clinicians is blood. However, it requires a high budget for training the personnel, collecting, storing and analyzing the samples, while the outputs consist of information on risk factors for several diseases. Epigenetic tests can assess the risks for metabolic, cardiovascular and immunologic diseases, as well as cancers and tumor recurrences.

A novel method with good results is the use of DBS [54]. It is a cost-effective method, as it requires less resources and the samples can be stored for years at room temperature. This method can be used for clinical interventions among the affected population, as well as for future research. A limitation of DBS is the fact that the RNA is not available in them. It can only be analyzed from frozen blood samples, implying large costs.

Human saliva as biologic material for epigenetic analysis is another cost-effective method [55]. It is easily collected with well-trained personnel and can be stored up to three weeks at room temperature. The DNA information available is similar, with some exceptions [55], and is on the same loci as the blood samples, offering great information on health risks. Keeping the saliva samples is, at the same time, not the best approach because of the large number of bacteria it contains and the risk for contamination. The DNA from it can be extracted and kept for future research. However, unlike genetic markers, epigenetic markers differ from tissue to tissue, and DNA extracted from saliva would offer different information than DNA extracted from blood samples or from biopsy samples.

As epigenetics is an evolving science and not all the transgenerational effects of famine have been discovered so far, so there is space for new outcomes. Scientists are working on discovering paths for diseases and collecting the proposed parameters at an early stage, which has a great value for future research. Applying this instrument in health prevention programs can bring prospective results and help health workers make early decisions and take specific actions to prevent these consequences.

### Strengths and Limitations

One limitation is the difficulty in disentangling epigenetic marks of famine from those of stress and harsh physical conditions, such as cold weather and intensive work. Stress accompanied famine in all studied cases. Efforts were made by the reviewers to narrow the list of selected studies to obtain a clear picture of the effects of famine.

Second, the epigenetic markers are still not clearly distinguished from the genetic ones, and the correlations discovered by now still need further research and confirmation.

This is the first review known so far that proposes a set of parameters to be assessed in disaster settings. Previous studies focused on epigenetic marks of famine, while this paper sums up the most relevant parameters and can be used in disaster contexts.

Future research on the transgenerational effects of famine is necessary, as well as on the value of parameters assessed for epigenetic markers. These studies will complete the list of proposed parameters, creating a manual for field workers and adding new insights on the data collected in emergency contexts.

## 5. Conclusions

Despite growing evidence of the most varied effects of famine on human health through epigenetic mechanisms, simple procedures with low cost could be undertaken at an early stage of famine to use for assessing the long-term impacts. This analysis shows that both laboratory methods, such as genetic and epigenetic testing, and quantitative assessment of exposure can be applied to routine programs. The epigenetic paths applied in epidemiology studies will help find correlations between exposure and disease. Developing and applying a set of parameters of exposure to calorie restriction would aid the risk assessment and disease prevention, as the effectiveness of the response depends on the availability of scientific evidence. The potential of the collected data is great with respect to policy making, operational and academic purposes.

## Figures and Tables

**Table 1:** The selected cohorts and the number of articles for each cohort.

Study Cohort	Context	Number of Articles
The Dutch Famine Birth Cohort	The German-occupied territory of Netherlands during the winter of 1944–1945	75
The Holocaust Survivors	More than 1000 ghettos in the eastern and central part of Europe, 1939–1945	448
The China’s Great Famine	China during The Great Leap, 1959–1961	22
The Siege of Leningrad	The German occupied city of Leningrad between 8 September 1941–1927 January 1944	16
Överkalix Cohort Study	Överkalix parish, Sweden, a sample from births in 1890, 1905, 1920, 1935	8
Biafran Study	The Nigerian Civil War, 1967–1970	5

**Table 2:** The health outcomes of famine exposure by the affected system.

Health Outcome Category	Examples
Metabolic	increased triacylglycerol concentration increased weight and body mass index metabolic syndrome impaired glucose tolerance and diabetes mellitus changes in dietary preferences, tendency to consuming high-fat diet osteoporosis with premature onset
Cardiovascular	ischemic heart disease cerebrovascular disease, stroke coronary artery disease with early onset increased blood pressure
Respiratory	asthma chronic obstructive pulmonary disease obstructive airways disease reduced lung function
Reproductive	impaired fecundity menstrual irregularities early onset of menopause
Neurological and psychiatric	smaller brain and intracranial volume congenital abnormalities schizophrenia and schizophrenia spectrum personality disorders changes in visual-motor skills, mental flexibility and selective attention
Oncologic	esophageal cancer gastric cancer colorectal cancer liver cancer breast cancer
Others	fatty liver disease increased levels of fibrinogen concentrations decreased levels of factor VII concentrations proteinuria microalbuminuria reduced creatinine clearance reduced Immunoglobulin E concentration

**Table 3:** Parameters to assess long-term effects of malnutrition (PALTEM) (in incremental order from low to high amount of financial resources needed).

Parameter	Qualification of the Personnel and Collecting Conditions	Storing Conditions	Level of Financial Resources Needed	Applications
Retrospective data collection:	Data abstractors: experience with retrospective data collection from clinical record, clinical and research experience, educational preparation in health care profession Training and orientation to the study protocol needed Data collection tools	Paper document or electronic record	Low	Assess the risk for adiposity in early childhood, metabolic diseases, especially obesity, impaired glucose tolerance (IGT), type 2 diabetes mellitus (T2DM), coronary arterial disease (CAD) Assess the risk for cardiovascular diseases (CVD)
Child’s birth weight and size				
Maternal pre-pregnancy BMI (ppBMI)				
Gestational weight gain (GWG)				
Mother’s glucose level				
Mother’s dietary habits: calorie intake, iron, fruits, preference for salty, fat food				
Clinical measurements:	Healthcare workers: clinical and research experience, educational preparation in health care profession	Paper document or electronic record	Low	Assess the risk for metabolic diseases, especially obesity, IGT, T2DM, CAD Assess the risk for CVD
Skinfold thickness				
Waist to hip ratio and BMI				
Heart rate (HR)				
Pulse wave velocity (PWV)				
Biological samples for post-hoc DNA methylation tests:	Healthcare workers: clinical and research experience, educational preparation in health care profession			Possible storage for years, enabling post-hoc analyses as knowledge evolves on specific DNA methylation sites. Assess the risk for metabolic diseases and pancreatic islets function; CVD and risk for stroke; Immunoglobulin E (IgE) concentration, osteoarthritis (OA) and rheumatoid arthritis (RA); cancer, tumor recurrence, survival and response to chemotherapeutic strategy
Buccal swabs of saliva	Buccal swab pouches	−20 °C to 25 °C, up to 3 weeks	Low	
Dried blood samples (DBS)	Whatman 903 filter cards, 3.2 mm punch	Room temperature, for years	Low	
Peripheral blood samples	Needles and syringes, tubes containing sterile EDTA solution	4 °C, −20 °C, or −80 °C, with or without 10% DMSO, for the following time periods: overnight (i.e., 15 h), 72 h, 1 week, or 1 month	High	

## Appendix A

### Appendix A.1. The Dutch Famine Birth Cohort

The Dutch Famine Birth Cohort is a pioneer of the historical cohort research design. It is based on very well delimited, with respect to time and space, circumstances created by the German blockade during the winter of 1944–1945 in the German-occupied territory of Netherlands. The blockade cut off the food and fuel supply, and because of the harsh weather conditions, delivery through the North Sea was not possible, as it was frozen. The adult ratios fell to less than 1000 kilocalories per day by the end of November 1944 and less than 580 kilocalories by the end of February 1945. The Netherlands did not face food scarcity before the Blockade, and shortly after the deliberation by the Allies, it started very shortly to receive relief, so the ratios returned to normal size very fast. Records from Wilhelmina Gasthuis hospital were used in order to create a cohort of 1380 live-born singleton born between 1 November 1944 and 28 February 1946, 650 live-born singletons born in the year before this period and of 650 live-born singletons born in the year thereafter. Of these 2680 babies, 27 (1%) were excluded because their birth record was missing and 239 (9%) were excluded because their gestational age at birth was <259 days, either as computed from the date of the last menstrual period or as estimated by the obstetrician at the first prenatal visit and at the physical examination of the baby just after birth. In all, 2414 live-born singletons were included [56]. The Population Registry of Amsterdam traced 2155 of these babies and due to death or emigration, the cohort number decreased to 1527. From the remaining cohort, 912 of people were invited for both interview and clinic attendance and only 741 of them were followed-up. The circumstances of famine and the well documented birth records allowed the researchers to categorize 3 periods of 16 weeks to distinguish between babies who were exposed during late gestation (born 7 January to 28 April), mid gestation (29 April to 18 August), and early gestation (19 August to 8 December) [56]. Anthropometric measures were performed and correlations between birth weight and current health status, both perceived and clinically assessed, were analyzed. Now investigations continue, including the second generation of offspring, so the traces of epigenetic marks can be monitored trans-generationally.

### Appendix A.2. The Holocaust Survivors

The Holocaust is another history event that offered circumstances to study the effects of famine and stress on fetus and infants. Between 1939 and 1945, more than 1000 ghettos were created mostly in the eastern and central part of Europe. The living conditions were brutal and varied in each country. Jews were forced to live in specific areas of the city, which were very small, so they were from 7 to 30 people in one room with very poor sanitation. They were not allowed to go outside the area, so the only food source consisted of the supplies from Nazis, which were very scarce: around 250 kilocalories per person per day.

Jews that survived the Holocaust and returned to the actual State of Israel were interviewed and health outcomes were assessed by clinical measurements. It is assumed that the living conditions were much better in the present State of Israel, so the population who migrated there did have enough food and satisfactory health care. Even the records were not so well documented as for the previous mentioned cohort, important investigations were performed. The cohort is mainly composed of the exposed group, that is people who migrated to Israel after the Second World War (258,048), and non-exposed group, those who migrated from Europe to Israel before or during the Second World War (57,496). As there is no individual record, the exposed group was based on the immigration records for Israeli born in Europe.

### Appendix A.3. The China’s Great Famine

The largest famine in human history was registered in China during 1959–1961 [57]. It was a manmade catastrophe, as a result of Mao Zedong’s decision to launch the Great Leap Forward, which was supported by the Communist Party. The plan was to industrialize the economy of the country, so tens of millions of peasants were forced to leave the work on fields and to move to cities. The villages were the most affected, as the government focused all attention and efforts towards cities. Emigration was strictly controlled and prohibited even between villages. As famine was a social catastrophe and a result of the governmental failure, statistical reports were fabricated and it is almost impossible to have a coherent picture of the morbidity, mortality and nutritional status. The most accurate demographic reconstruction indicates an estimation of 30 million dead [58]. The famine lasted two years and the situation after it did not undergo a rapid improvement. The exposed group consists of those born during and immediately after famine, so they were exposed both pre- and post-natal.

### Appendix A.4. The Siege of Leningrad

The Siege of Leningrad was a period of 872 days, in which the German occupied city was blocked by the German and Finnish armies. The Red Army tried to build antitank fortifications, but soon the city was completely encircled and no rail or other supply lines could reach it, leaving the population without any food supply during harsh winter conditions. From 20 November 1941 the only available food was 125 g of bread per day for civilians and 250 g for manual workers. Children over 12 years received even less—only 200 g of fat, 800 g of sugar, and 600 g of carbohydrate a month [59]. After the successful offensive of the Soviet army in January 1944, the siege was ended. Notwithstanding, the situation did not change dramatically. The circumstances after the war made it difficult for the citizens to recover and return to a normal healthy life.

In a study conducted in 2004, 5000 men born in 1916–1935 were randomly selected from voting lists in the socially mixed Petrogradsky district and 3905 of them (78%) participated. 1406 lived in Leningrad during the siege, which meant that they spent the whole siege period there, as most people were unable to leave [28]. Death certificates were obtained to confirm the cause of death, which was coded according to ICD-8. The survivors had their vital status ascertained through direct contact.

### Appendix A.5. The Överkalix Study

The Överkalix Study was conducted to assess longevity and causes of death among the probands born in Överkalix parish, northernmost Sweden. Information on harvest and food prices was estimated and recorded by a 19th century statistician [60], and it was used to assess food availability to the probands’ parents and grandparents [61]. Usually a year rich in harvest was followed by one in which the availability of food was scarce and given the location of the municipality, it could not be reached neither by any transportation available in the 19th century, nor by sea, as it was frozen.

A sample from births in 1890, 1905, 1920 and 1935, the years when the harvest reached the lowest peaks, was selected. Parents’ and grandparents’ food availability at critical age 9–12 years was defined by regional data on harvest and food prices over the period 1803–1849 using a three point scale (good, intermediate, poor) [61]. A 50% sample of the 319 individuals born in Överkalix in the selected years and 1626 matched parents and grandparents was created, excluding those not traced, probands with missing data on childhood or ancestors with unknown birth dates [61]. The study was aimed to determine the cause of death in the third generation.

### Appendix A.6. The Biafran Study

The Biafran Study is an observational study based on a cohort of 1399 subjects from the igbo group, born during 1965–1973, a period before, during and after famine [62]. The Nigerian Civil War started in July 1967 and only 10% of deaths were due to violence, the rest of them were a result of famine. Around seven million people were affected by famine and the first international aid that came one year after the beginning of the war did only reach a small number of them [62]. The subjects were categorized in three groups: exposed to famine in early childhood, exposed to famine in fetal and infant life and those unexposed [62]. The aim of the study was to determine the risk for hypertension, glucose intolerance and overweight after exposure to famine in utero.

## Appendix B

**Table A1:** Description of the included studies, the variables and parameters collected and the main findings (*p*-value = probability value, SD = standard deviation, CI = confidence interval, *n* = number of subjects).

First Author, Year	Cohort Description	Aim of Study	Collected Variables and Parameters	Outcomes and Effect Size
The Dutch Famine Birth Cohort				
Stein, 1975 [63]	Seven cohorts of unequal size, based on the criterion of stage of gestation in relation to famine exposure: August–October 1944 (conceived and born before famine); November–January 1944–1945 (exposed at the third semester); Februar–April 1945 (exposed at the second and third trimester); May–June 1945 (exposed to famine during the middle 6 months of gestation); July–September 1945 (exposed during first and second trimester of gestation); October–January 1945–1946 (exposed during the first semester); Februar–March 1946 (conceived and born after the famine).	To study the effects of famine during pregnancy on six indices at birth of the newborns.	*Independent variables:* - Maternal characteristics: age, primipara, manual class; - Stage of gestation, in relation to famine exposure; - Rations of calories, fats, proteins, carbohydrates on averages for the exposure period. *Outcome parameters:* - Birth weight; - Placental weight; - Infant length; - Head circumference; - Duration of gestation; - Maternal weight.	- Birth weight (g): Amsterdam (mean = 3211, SD = 665, *n* = 1148), Leiden (mean = 3331, SD = 607, *n* = 457), Rotterdam (mean = 3245, SD = 654, *n* = 806), Groningen (mean = 3311, SD = 659, *n* = 1290), Heerlen (mean = 3345, SD = 551, *n* = 1149); - Placental weight (g): Rotterdam (mean = 573, SD = 130, *n* = 775), Groningen (mean = 641, SD = 147, *n* = 1236), Heerlen (mean = 575, SD = 160, *n* = 1097); - Infant length (cm): Amsterdam (mean = 49.0, SD = 4.1, *n* = 973), Leiden (mean = 50.0, SD = 3.9, *n* = 457), Rotterdam (mean = 49.6, SD = 3.6, *n* = 794), Groningen (mean = 49.3, SD = 3.5, *n* = 1272), Heerlen (mean = 49.6, SD = 2.8, *n* = 1129); - Head circumference (cm): Rotterdam (mean = 34.9, SD = 2.1, *n* = 644), Groningen (mean = 33.7, SD = 1.5, *n* = 1197), Heerlen (mean = 35.1, SD = 1.7, *n* = 760); - Duration of gestation (weeks): Amsterdam (mean = 39.4, SD = 2.3, *n* = 1144), Rotterdam (mean = 39.4, SD = 2.9, *n* = 791), Groningen (mean = 39.5, SD = 2.4, *n* = 1021), Heerlen (mean = 39.5, SD = 2.5, *n* = 1085); - Maternal weight (kg): raw data available only for births in Rotterdam, difficult to estimate the reliability of the results.
Lopuhaa, 2000 [30]	Five cohorts of unequal size, based on the criterion of stage of gestation in relation to famine exposure: 264 born before famine 1 November 1943 to 6 January 1945); 140 exposed to famine in late gestation 29 April 1945 to 18 August 1945); 137 exposed to famine in mid gestation (29 April 1945 to 18 August 1945); 87 exposed to famine in early gestation (19 August 1945 to 8 December 1945); 284 conceived after famine (9 December 1945 to 28 February 1947 ).	To study the effects of famine during pregnancy on the prevalence of the obstructive airways disease and atopy in the first generation.	*Independent variables:* - Stage of gestation, in relation to famine exposure; - Rations of calories, fats, proteins, carbohydrates on averages for the exposure period. - Maternal characteristics: age, primipara, weight at the end of pregnancy, manual class; - Characteristics at age 50: current smoker, socioeconomic status. *Outcome parameters:* - Birth characteristics: weight, body length, head circumference, ponderal index, gestational age. - Characteristics at age 50: weight, height; - Total immunoglobulins E (IgE); - Lung function: forced expiratory volume(FEV), forced vital capacity (FVC), FEV/FVC; - Respiratory symptoms and disease: wheeze, productive cough, obstructive airway disease (OAD).	- Total IgE (IU/mL): mean = 30.4, SD = 4.6, *n* = 726; - Lung function (L: *n* = 733, FEV (L): mean = 3.1, SD = 0.6, FVC (L): mean = 4.3, SD = 0.7, FEV/FVC: mean = 0.72, SD = 0.1; - Respiratory symptoms and disease: *n* = 912, wheeze 11.7%, productive cough 4.5%, OAD 18.1%.
Roseboom, 2000 [64]	Five cohorts of unequal size, based on the criterion of stage of gestation in relation to famine exposure: 264 born before famine (1 November 1943 to 6 January 1945); 140 exposed to famine in late gestation (7 January 1945 to 28 April 1945); 137 exposed to famine in mid gestation (29 April 1945 to 18 August 1945); 87 exposed to famine in early gestation (19 August 1945 to 8 december 1945); 284 conceived after famine (9 December 1945 to 28 February 1947).	To study the effects of famine during pregnancy on the prevalence of coronary heart disease in first generation.	*Independent variables:* - Stage of gestation, in relation to famine exposure; - Rations of calories, fats, proteins, carbohydrates on averages for the exposure period. - Maternal characteristics: age, primipara, weight at the end of pregnancy, manual class; - Smoking status; - Alcohol habits; - Socioeconomic status (SES). *Outcome parameters:* - Birth characteristics: weight, body length, head circumference, ponderal index, gestational age. - Adult characteristics: body mass index (BMI), systolic blood pressure (SBP), glucose 20 min, low density lipoproteins (LDL), high density lipoprotein (HDL); - Prevalence of coronary heart disease (CHD).	- Prevalence of CHD: 3.3%, SD = 24, *n* = 736; - BMI (kg/m^3^): mean = 27.0, SD = 1.2, *n* = 736; - SBP (mmHg): mean = 126, SD = 16, *n* = 734; - Glucose 120 min (mmol/L): mean = 6.0, SD = 1.4, *n* = 697; - LDL: HDL cholesterol (mmol/L): mean = 2.9, SD = 1.5, *n* = 697; - SES: mean = 48, SD = 13, *n* = 736; - Smoking: 34%, *n* = 736; - Alcohol (units/day): mean = 9, SD = 11, *n* = 736.
Fransen, 2016 [65]	The Prospect-EPIC cohort [66], categorized as [67]: Unexposed (3450); Moderately exposed (2838); Severely exposed (1237). Total: 7527 women.	To investigate the association between exposure to famine in childhood and adolescence and unhealthy lifestyle later in life for the first generation.	*Independent variables:* - Age at start of famine; - Period of exposure to famine; - Age at recruitment. *Outcome parameters:* - BMI; - Waist; - Level of education; - Smoking status; - Alcohol consumption; - Energy intake; - Modified Mediterranean diet score (mMDS); - Unhealthy diet; - Physical inactivity.	- Prevalence of smoking: 1.10 (95% CI, 1.05; 1.14) for moderately exposed and 1.18 (95% CI, 1.12; 1.25) for severely exposed women (*p*-value < 0.0001); - Pack years of smoking: moderately exposed women reported smoking 0.98 (95% CI, 0.10; 1.87) more pack years than unexposed women, while severely exposed women reported 2.53 (95% CI, 1.39; 3.66) more pack years ( *p*-value < 0.0001); - Prevalence of heavy drinking: moderately: 0.94 (95% CI, 0.85; 1.03) and severely exposed women: 0.95 (95% CI, 0.84; 1.07), compared to unexposed women (*p* = 0.04); - Unhealthy diet: prevalence ratio 0.92 (95% CI, 0.86; 0.98) for moderately exposed to unexposed, no differences between severely exposed and unexposed (*p* = 0.51); - mMDS: moderately exposed women had a 0.08 point (95% CI: 0.00; 0.16) higher mMDS than unexposed women, no differences between severely exposed and unexposed (*p* = 0.77); - Physical inactivity: prevalence ratio for moderately exposed: 1.18 (95% CI, 0.99; 1.42) and severely exposed: 1.32 (95% CI, 1.06; 1.64) (*p* for trend = 0.08).
Elias, 2003 [68]	The Prospect-EPIC cohort [66], categorized as [67]: Unexposed (45); Moderately exposed (28); Severely exposed (14). Total: 87 women.	To investigate the consequences of childhood exposure to famine on the insulin-like growth factor axis in first generation.	*Independent variables:* - Age during famine; - Age at recruitment; - Smoking status; - Alcohol habits. *Outcome parameters:* - BMI; - Waist; - Insulin-like growing factor I (IGF-I); - Insulin-like growing factor binding protein (IGFBP)-1,2,3; - Connecting peptide (C-peptide).	- IGF-I (ng/mL) (mean (95% CI)): unexposed: 132.9 (121.4–145.6), moderately exposed: 142.0 (1126.6–159.3), severely exposed: 162.3 (138.0–191.0), *p*-value = 0.038; - IGFBP-1 (ng/mL) (mean (95% CI)): unexposed: 12.6 (10.0–16.0), moderately exposed: 10.2 (7.5–13.8), severely exposed: 9.6 (6.2–14.7), *p* value = 0.179; - IGFBP-2 (ng/mL) (mean (95% CI)): unexposed: 388.2 (341.2–441.7), moderately exposed: 335.4 (284.7–395.2), severely exposed: 344.6 (270.8–438.7), *p*-value = 0.219; - IGFBP-3 (ng/mL) (mean (95% CI)): unexposed: 3170.7 (3029.0–3319.1), moderately exposed: 3236.9 (3053.1–3431.7), severely exposed: 3511.8 (3235.6–3811.7), *p*-value = 0.045; - C-peptide (ng/mL) (mean (95% CI)): unexposed: 3.3 (2.8–3.8), moderately exposed: 3.3 (2.7–4.1), severely exposed: 3.2 (2.4–4.3), *p*-value = 0.946.
Painter, 2005 [44]	Five cohorts of unequal size, based on the criterion of stage of gestation in relation to famine exposure: 264 born before famine (1 November 1943 to 6 January 1945); 140 exposed to famine in late gestation (7 January 1945 to 28 April 1945); 137 exposed to famine in mid gestation (29 April 1945 to 18 August 1945); 87 exposed to famine in early gestation (19 August 1945 to 8 December 1945); 284 conceived after famine (9 December 1945 to 28 February 1947).	To study the effects of famine during pregnancy on the prevalence of microalbuminuria in first generation.	*Independent variables:* - Stage of gestation, in relation to famine exposure; - Rations of calories, fats, proteins, carbohydrates on averages for the exposure period; - Smoking status; - Alcohol habits; - SES. *Outcome parameters:* - Birth outcomes (weight, length, placental area); - Maternal characteristics (weight at last prenatal visit); - Adult characteristics (BMI, SBP, diastolic blood pressure (DBP), total cholesterol, impared glucose tolerance (IGT), electrocardiogram (ECG), creatinine clearance, hemoglobin alpha 1 (HbA1).	Odds ratio (OR) for albumin-creatinine ratio (ACR) ≥ 2.5 compared with non-exposed group, adjusted for gender, age, adult BMI, smoking, SES (ISEI-92), SBP, IGT or non-insulin dependent diabetes mellitus (NIDDM) (2-h glucose > 7.8 or known diabetic), cholesterol, and ECG abnormalities: - Late exposure: 1.27 (0.49–3.26); - Mid exposure: 3.22 (1.34–7.65); - Early exposure: 1.89 (0.59–6.11).
Van Noord, 2004 [14]	Doorlopend Onderzoek Morbiditeit en Mortaliteit (DOM) cohort [69], categorized as [67] unexposed, moderately and severely exposed.	To explain the opposing effects of caloric deprivation during pregnancy and childhood on breast cancer and its risk factors in the first generation.	*Independent variables:* - Stage of gestation or age in relation to famine exposure; - Rations of calories, fats, proteins, carbohydrates on averages for the exposure period; *Outcome parameters:* - Age at menarche; - Adult height, weight, BMI, waist: hip ratio; - Age at menopause; - Mammographic density patterns and dysplasia; - Urinary postmenopausal hormones: estrone, estradiol, testosterone; - Plasma IGF-1 and IGFBP-3 levels.	Crude hazard ratio for breast cancer risk: - Unexposed: 1, number of cases = 265; - Moderately exposed: 1.17 (0.96–1.42), number of cases = 234; - Severely exposed: 1.54 (1.16–2.05), number of cases = 79.
Painter, 2006 [26]	Subjects exposed to the 1944–1945 Dutch famine during late (*n* = 160), mid- (*n* = 138), or early (*n* = 87) gestation and 590 unexposed subjects at age 50 or 58 year.	To investigate the early onset of coronary disease in first generation after prenatal exposure to famine.	*Independent variables:* - Stage of gestation, in relation to famine exposure; - Rations of calories, fats, proteins, carbohydrates on averages for the exposure period. - Maternal characteristics: age, primipara, weight at the end of pregnancy, manual class; - Smoking status; - Alcohol habits; - SES. *Outcome parameters:* - Birth characteristics: weight, body length, head circumference, ponderal index, gestational age. - Adult characteristics: BMI, SBP, glucose 120 min, LDL, HDL cholesterol; - Cumulative incidence of CAD.	Persons conceived during the famine were 3 years younger than the unexposed persons at the time of CAD diagnosis (47 years compared with 50 years) and had a higher cumulative incidence of CAD [13%; hazard ratio (HR) adjusted for sex: 1.9; 95% CI: 1.0, 3.8] than did the unexposed persons.
Stein, 2007 [70]	- exposed persons born in western Netherlands between January 1945 and March 1946 whose mothers experienced famine during or immediately preceding pregnancy (by ordinal weeks 1–10, 11–20, 21–30, and 31 through delivery) (356); - unexposed persons born in the same 3 institutions during 1943 or 1947 whose mothers did not experience famine during this pregnancy (296); - unexposed same-sex siblings of persons in series 1 or 2 (310).	To assess the relation between gestational exposure to famine and offspring length, weight, indexes of adiposity in middle age (for the first generation).	*Independent variables:* - Stage of gestation, in relation to famine exposure; - Maternal characteristics: age, primipara, weight at the end of pregnancy, manual class. *Outcome parameters (in adults):* - Height; - Trunk length; - Leg length; - Arm length; - Weight; - Waist circumference; - Hip circumference; - Supine sagittal abdominal diameter (SAD); - Midthigh circumference; - Subscapular skinfold thickness; - Triceps skinfold thickness; - Anterior midthigh skinfold thickness.	Exposure to starvation during gestation is strongly associated with a wide range of distribution of BMI among middle-age women (*p*-value < 0.05 for all, except the waist-to-hip ratio, for which *p*-value < 0.10). No other measures of length or body proportions in either men or women were associated with this condition.
De Rooij, 2007 [15]	Seven hundred and eighty-three subjects born before the famine (*n* = 238), exposed to famine in late gestation (*n* = 141), in mid gestation (*n* = 116), in early gestation (*n* = 74) and conceived after the famine (*n*= 214).	To determine the association between prenatal famine exposure and the prevalence of metabolic syndrome in first generation.	*Independent variables:* - Stage of gestation, in relation to famine exposure; - Rations of calories, fats, proteins, carbohydrates on averages for the exposure period. - Maternal characteristics: age, primipara, weight in 3rd trimester and at the end of pregnancy, manual class; - Smoking status; - Sport practice; - Alcohol habits; - SES; - Current age. *Outcome parameters (in adults):* - Adult characteristics: BMI, SBP, glucose 120 min, LDL, HDL cholesterol, waist circumference, blood pressure.	Exposure to famine during gestation was not significantly associated with the metabolic syndrome (OR: 1.2; 95% CI: 0.9, 1.7). Birth weight also was not significantly associated with the metabolic syndrome (OR: 1.3/1-kg decrease in birth weight; 95% CI: 0.9, 1.8/1-kg decrease in birth weight). Exposure to famine during gestation was associated with significantly higher triacylglycerol concentrations (0.1 g/L; 0.0, 0.2 g/L). Men exposed to famine in early gestation had significantly lower HDL-cholesterol concentrations (−0.08 mmol/L; −0.14, 0.00 mmol/L) than did unexposed men
Painter, 2008 [71]	Eight hundred and fifty-five subjects: 264 unexposed and born before famine, 350 prenatally exposed to famine, 242 unexposed and conceived after famine; 1496 subjects of the second generation (F2), accordingly to the exposure status of the first generation (F1).	To assess the effects of prenatal exposure to famine on neonatal adiposity and health in later life.	*Independent variables:* - Stage of gestation, in relation to famine exposure; - Maternal characteristics: age, primipara, weight in 3rd trimester and at the end of pregnancy, manual class; - Smoking status; - SES; - Current age. *Outcome parameters (in adults):* - Birth characteristics: weight, body length, head circumference, ponderal index, gestational age. - Adult characteristics: age, BMI, waist circumference, SBP, glucose 120 min, LDL, HDL cholesterol; - Number of offspring.	F2 birth length was decreased (−0.6 cm, *p* adjusted for F2 gender and birth order = 0.01) and F2 ponderal index was increased (+1.2 (kg/m^3^), *p* adjusted for F2 gender and birth order = 0.001). F1 women exposed to famine in utero also responded that had poor health 1.8 (95% CI, 1.1–2.7) times more frequently in later life than that of F1 unexposed women.
Van Hoek, 2009 [72]	Seven hundred and seventy-two subjects born before the famine (*n* = 233), exposed to famine in late gestation (*n* = 140), in mid gestation (*n* = 117), in early gestation (*n* = 71) and conceived after the famine (*n* = 211).	To investigate the effects of fetal malnutrition on type 2 diabetes risk and related phenotypes in first generation of offspring.	*Independent variables:* - Stage of gestation, in relation to famine exposure; - Maternal characteristics: age, primipara, weight in 3rd trimester and at the end of pregnancy, manual class; - Smoking status; - SES; - Current age. *Outcome parameters (in adults):* - Birth characteristics: weight, body length, head circumference, ponderal index, gestational age. - Adult characteristics: age, BMI, waist circumference; - Fasting blood samples for genomic analyses of rs7754840 (*CDKAL1*), rs10811661 (*CDKN2AB*), rs1111875 (*HHEX*), rs4402960 (*IGF2BP2*), rs5219 (*KCNJ11*), rs13266634 (*SLC30A8*), and rs7903146 (*TCF7L2*) polymorphisms.	The *TCF7L2* and *IGF2BP2* variants were associated with increased type 2 diabetes mellitus (T2DM)/IGT risk (TCF7L2: OR 1.39 [95% CI 1.08–1.79], *IGF2BP2*: 1.43 [1.11–1.85]) and increased area under curve (AUC) for glucose (*TCF7L2*: β = 4.5 [1.0–8.1], IGF2BP2: β = 3.6 [0.1–7.1]). The *CDKAL1* variant associated with a decreased AUC for insulin (β = −8.2 [−16.1 −0.41]), which became less strong after adjustment for BMI. The IGF2BP2 showed a significant interaction on AUC glucose (β interaction −9.2 [−16.2 −2.1], *p*-value = 0.009). None of the polymorphisms was associated with birth weight.
Haars, 2010 [73]	One thousand and thirty-five women from DOM project [69]: 452 unexposed to the famine, 358 moderately exposed and 225 severely exposed.	To examine how breast density is affected by short caloric restriction in childhood and adulthood, and whether the effect is dependent on the exposure age.	*Independent variables:* - Age at exposure to famine; - Age at mammography; - Height, weight, BMI; - Menopausal status; - Parity. *Outcome parameters (in adults):* - Breast size; - Breast density (mammography).	In unexposed compared to severely exposed women, means varied from 124 cm^2^ to 121 cm^2^ (*p*-value = 0.50) for breast size, from 23.4 to 21.8 cm^2^ (*p*-value = 0.48) for amount of dense tissue, from 87.7 to 85.4 cm^2^ (*p*-value = 0.55) for non-dense tissue and from 22.8 to 22.3% (*p*-value = 0.78) for relative density. Only among women who were younger than 10 years during the famine was the amount of non-dense tissue significantly lower with higher exposure, with 53.1 cm^2^ for severely exposed compared to 77.8 cm^2^ (*p*-value = 0.03) for unexposed.
Botden, 2012 [74]	Seven hundred ninety-three individuals born as term singletons in Amsterdam around the famine in the Netherlands during World War II, as described in detail earlier [75].	To investigate whether Sirutin 1 (SIRT1) influences fetal programming during malnutrition.	*Independent variables:* - Period of exposure to famine. *Outcome parameters (in adults):* - Fasting glucose level; - Oral glucose tolerance test (OGTT); - AUC for glucose and insulin; - BMI; - Blood samples for SIRT1 genetic investigation.	A significant interaction was found between two SIRT1 single nucleotide polymorphisms (SNPs) and exposure to famine in utero on T2DM risk (*p*-value = 0.03 for rs7895833; *p*-value = 0.01 for rs1467568). Minor alleles of these SNPs were associated with a lower prevalence of T2DM only in individuals who had been exposed to famine prenatally (OR for rs7895833 0.50 [95% CI 0.24–1.03], *p*-value = 0.06; for rs1467568 0.48 [0.25–0.91], *p*-value = 0.02).
Van Abeelen, 2012 [76]	Seven thousand five hundred and fifty-seven women from Prospect-EPIC cohort [66], with the exposure age classified into three categories: childhood (age 0–9 years), adolescence (age 10–17 years), and young adulthood (age ≥18 years) [67].	To investigate the association between childhood and adulthood undernutrition and T2DM in adulthood.	*Independent variables:* - Period of exposure to famine. *Outcome parameters (in adults):* - Weight, height, BMI; - Waist: hip ratio; - Urinary samples for glucose strip test.	For moderate famine exposure, the age-adjusted T2DM HR was 1.36 (95% CI [1.09–1.70]); for severe famine exposure, the age-adjusted HR was 1.64 (1.26–2.14) relative to unexposed women.
Tobi, 2012 [77]	One hundred and twenty individuals: 60 (28 males and 32 females, age at examination 58.1 year [SD, 0.35 year]) exposed to famine around the moment of conception and first 10 weeks of gestation; 24 same-sex siblings (11 male, 13 female) conceived and born before the famine; 36 same-sex siblings (17 male, 19 female) conceived and born after the famine. Age at examination 57.0 year [SD, 5.9 year].	To test if the associations between famine exposure and genetic variation are independent and to contrast the effect sizes of these associations.	*Independent variables:* - Period of exposure to famine. *Outcome parameters (in adults):* - Blood samples for analysis of IGF2/H19 methylation.	The average deoxyribonucleic acid (DNA) methylation difference between exposed and unexposed was 0.5 SD for significantly associated differentially methylated regions (DMRs). Only the interactions between prenatal famine exposure and INSIGF SNPs rs3842756 (*p* = 0.048) and rs689 (*p* = 0.016) in relation to IGF2 DMR1 methylation were significant.
Tobi, 2015 [78]	Three hundred and forty-eight exposed to famine in utero: 73 in weeks 1–10 of gestation; 123 in weeks 11–20 of gestation; 143 in weeks 21–30 of gestation; 128 in weeks 31-delivery. (Some individuals meet the definition for exposure in two adjacent gestation periods.) 160 time-controls (1943–1947). 303 same-sex siblings.	To study the epigenome-wide association for famine exposure during specific gestation periods and for exposure to famine in any period during gestation.	*Independent variables:* - Stage of gestation, in relation to famine exposure; - Rations of calories, fats, proteins, carbohydrates on averages for the exposure period. - Smoking habits; - Dietary intake of micro- and macronutrients; - SES. *Outcome parameters (in adults):* - Blood samples to study the DNA methylation.	Famine exposure during gestation weeks 1–10, but not weeks 11–20, 21–30 or 31-delivery, was associated with an increase in DNA methylation of cytosine phosphate guanine (CpG) dinucleotides cg20823026 (FAM150B), cg10354880 (SLC38A2) and cg27370573 (PPAP2C) and a decrease of cg11496778 (OSBPL5/MRGPRG) (*p* < 5.9 × 10^−7^, positive false discovery rate (PFDR) <0.031). There was an increase in methylation of TACC1 and ZNF385A after exposure during any time in gestation (*p* < 2.0 × 10^−7^, PFDR = 0.034) and a decrease of cg23989336 (TMEM105) after exposure around conception. SD: 0.3–0.6.
De Rooij, 2016 [34]	One hundred and eighteen subjects: 41 exposed to famine in early gestation; 77 unexposed to famine in gestation. Mean age: 67.5 year.	To assess the effects of undernutrition during early gestation on brain size, structure, and white matter integrity at 68 year.	*Independent variables:* - Stage of gestation, in relation to famine exposure; - Rations of calories, fats, proteins, carbohydrates on averages for the exposure period. - Maternal characteristics: age, primipara, weight in 3rd trimester and at the end of pregnancy, manual class; - Smoking status; - Sport practice; - Alcohol habits; - SES; - Current age. *Outcome parameters (in adults):* - Adult characteristics: BMI, SBP, glucose 120 min, LDL, HDL cholesterol, waist circumference, blood pressure. - Magnetic Resonance Imaging (MRI) scan: T1 structural scan; Diffusion Tensor Imaging (DTI) scan; Fluid-attenuated inversion recovery (FLAIR) scan.	Intracranial volume (ICV) and total brain volume (TBV) were larger in males than in females [119 mL (95% CI: 88–150) and 116 mL (86–146)]. Birth weight, head circumference at birth and at age 68 were all significantly positively associated with ICV and TBV (all *p* < 0.05). Males exposed to famine during early gestation had smaller ICV than unexposed males with a mean difference of 58 mL (98% CI: 11–106), corresponding to a difference of ∼5%.
Roseboom, 2000 [42]	Seven hundred and twenty-five subjects: 209 born before famine; 117 exposed to famine in late gestation; 41 exposed to famine in mid-gestation; 65 exposed to famine in early gestation; 226 conceived after famine.	To assess the effect of maternal malnutrition on plasma fibrinogen and factor VII concentrations in first generation adults.	*Independent variables:* - Stage of gestation, in relation to famine exposure; - Rations of calories, fats, proteins, carbohydrates on averages for the exposure period. - Maternal characteristics: age, primipara, weight in 3rd trimester and at the end of pregnancy, manual class; - Smoking status; - Sport practice; - Alcohol habits; - SES; - Current age. *Outcome parameters (in adults):* - Adult characteristics: BMI, SBP, glucose 120 min, LDL, HDL cholesterol, waist circumference, blood pressure. - Plasma fibrinogen; - Factor VII.	Plasma fibrinogen concentrations differed by −0.01 g/L (95% CI, −0.14–0.11) in those exposed in late gestation, by −0.03 g/L (95% CI, −0.16–0.11) in those exposed in mid gestation, and by 0.13 g/L (95% CI, −0.03–0.30) in those exposed in early gestation, compared with non-exposed people. Plasma factor VII concentrations differed by 0.4% (95% CI, −5.4%–6.6%) in those exposed to famine in late gestation, by 1.5% (95% CI, −4.6%–8.1%) in those exposed in mid gestation. and by −11.8% (95% CI, −18.4–−4.8%) in those exposed in early gestation, compared with non-exposed people.
The Chinese Famine Cohort				
Li Y, 2011 [17]	Seven thousand eight hundred and seventy-four individuals From severely affected famine area: 834 non-exposed, 334 fetal exposed, 641 early childhood exposed, 630 mid-childhood exposed, 613 late childhood exposed; From less severely affected famine area: 1120 non-exposed, 671 fetal exposed, 1013 early childhood exposed, 958 mid-childhood exposed, 1060 late childhood exposed.	To examine if there is any association between fetal exposure to famine and the risk of metabolic syndrome in later life.	*Independent variables:* - Age period for famine exposure; - Severity of famine in the area. *Outcome parameters (in adults):* - Weight; - Height; - Waist circumference; - Blood pressure; - Fasting plasma glucose (FPG); - Blood samples for OGTT; - Blood samples for LDL, HDL, triglyceride (TG) plasma concentrations.	Severely affected famine area (prevalence, OR, 95% CI, *p* of metabolic syndrome): - Non-exposed: 3.1%, 1.0; - Fetal exposed: 7.8%, 3.13, [1.24–7.89], *p* = 0.016; - Early childhood exposed: 7.1%, 2.85, [1.19–6.83], *p* = 0.019; - Mid-childhood exposed: 5.7%, 2.07, [0.77–5.53], *p* = 0.15; - Late childhood exposed: 6.4%, 2.21, [0.91–5.40], *p* = 0.08. Less severely affected famine area (prevalence, OR, 95% CI, *p* of metabolic syndrome): - Non-exposed: 9.4%, 1.0; - Fetal exposed: 7.4%, 0.80, [0.46–1.41], *p* = 0.44; - Early childhood exposed: 9.7%, 0.91, [0.55–1.51], *p* = 0.72; - Mid-childhood exposed: 10.1%, 0.97, [0.57–1.64], *p* = 0.91; - Late childhood exposed: 11.6%, 1.29, [0.76–2.19], *p* = 0.34.
Li QD, 2012 [79]	Birth cohorts who were exposed to the 1959–1961 Chinese famine and birth cohorts who were not exposed.	To describe the stomach cancer mortality trends in different cohorts that had been exposed to long-term malnutrition during early life.	*Independent variables:* - Age period for famine exposure; - Severity of famine in the area. *Outcome parameters (in adults):* - Data of stomach cancer mortality.	For males: relative risk (RR) 2.39, 95% CI 1.51–3.77. For females: RR 1.64, 95% CI 1.02–2.62.
Wang PX, 2012 [28]	Twelve thousand and sixty-five subjects born in Nanhai and Zhongshan areas in 1957–1964.	To assess the impact of exposure to the 1959–1961 Chinese Great Famine during fetal development and first 2 years of postnatal life on the risk of hypertension, short stature and obesity in adulthood.	*Independent variables:* - Age period for famine exposure; - Severity of famine in the area; - Questionnaire to assess: sex, age, occupation, smoking, alcohol use, regular physical activity, preference for salty foods, municipality, place of residence. *Outcome parameters (in adults):* - Weight; - Height; - Blood pressure.	Subjects exposed during the 1st trimester only had significantly higher SBP, DBP and risk of hypertension [adjusted OR = 1.36 (1.03, 1.79)]. The risk of hypertension was about 1.8-fold higher in subjects exposed to famine during infancy only (*p* < 0.001), and 1.3-fold higher in subjects exposed during both fetal development and infancy (*p* < 0.001), but was not significantly elevated in those exposed during fetal development only overall (*p* = 0.15).
Shi, 2013 [80]	Two thousand and seven subjects born between 1952 and 1964, from Jiangsu province, China.	To investigate if early life exposure to famine is related to higher risk of anemia in adulthood.	*Independent variables:* - Age period for famine exposure; - Severity of famine in the area; - Questionnaire to assess: sex, age, occupation, smoking, alcohol use, regular physical activity, preference for salty foods, municipality, place of residence; - Nutrients intake using a 3-day weighted food diary. *Outcome parameters (in adults):* - Weight; - Height; - Blood pressure; - Overnight fasting blood samples for hemoglobin (cyanmethemoglobin method).	Prevalence of anemia in adulthood: - non-exposed: 26.0%, - fetal-exposed: 33.8%, - early-childhood: 28.1%, - mid-childhood: 28.2%, - late-childhood: 29.7%. RR for fetal-exposed to famine (95% CI) (RR (95% CI)) was 1.37 (1.09, 1.71).
Huang, 2014 [43]	Seventy thousand five hundred and forty-three women born between 1957–1965, in Zhejiang Province, China.	To investigate the associations between early life exposure to the 1959–1961 Chinese Great Famine and the levels of protein in urine in adulthood.	*Independent variables:* - Age period for famine exposure; - Severity of famine in the area; - Questionnaire to assess: sex, age, occupation, smoking, alcohol use, regular physical activity, preference for salty foods, municipality, place of residence. *Outcome parameters (in adults):* - Weight; - Height; - Blood pressure; - Urine samples for protein concentration.	Famine exposure and levels of proteinuria in the rural sample (mg/day), (*n*= 51,978, OR (95% CI), *p*-value): Pre-famine cohort: 1.28 (0.73, 2.25), 0.366; Famine cohort: 1.53 (1.04, 2.16), 0.031; Post-famine cohort: 1.26 (0.99, 1.59), 0.052. Famine exposure and levels of proteinuria in the urban sample (mg/day), (*n* = 4563, OR (95% CI), *p*-value): Pre-famine cohort: 0.63 (0.18, 2.21), 0.471; Famine cohort: 0.90 (0.36, 2.28), 0.824; Post-famine cohort: 1.17 (0.65, 2.10), 0.610.
Wang, 2015 [18]	Six thousand eight hundred and ninety-seven adults from East China, Shanghai and 7 provinces: 1245 non-exposed (born after 1975); 1808 non-exposed born between 1963–1974); 745 fetal-exposed (1959–1962); 1911 childhood-exposed (1949–1958); 1188 adolescence/adult-exposed (1921–1948).	To explore whether early life exposure to famine and high economic status in adulthood is associated with diabetes in later life.	*Independent variables:* - Age period for famine exposure; - Severity of famine in the area; - Questionnaire to assess: sex, age, occupation, smoking, alcohol use, regular physical activity, preference for salty foods, municipality, place of residence. *Outcome parameters (in adults):* - Weight; - Height; - Blood pressure; - Venous blood samples for plasma glucose test, HbA1C, fasting plasma glucose (FPG), lipid profile (cholesterol, TG, HDL, LDL, insulin secretion, insulin resistance).	Exposure to starvation in utero, associated with a high economic status in adult life increases the prevalence of diabetes in middle ages and old ages. Famine exposure during the fetal period (OR 1.53, 95% CI 1.09–2.14) and childhood (OR 1.82, 95% CI, 1.21–2.73) was associated with diabetes. Subjects living in areas with high economic status had a greater diabetes risk in adulthood (OR 1.46, 95% CI 1.20–1.78). In gender-specific analyses, fetal-exposed men (OR 1.64, 95% CI, 1.04–2.59) and childhood-exposed women (OR 2.81, 95% CI, 1.59–4.97) had significantly greater risk of diabetes.
The Siege of Leningrad Survivors (prospective cohort study, St. Petersburg, Russia)				
Sparen, 2004 [24]	Three thousand nine hundred and seven men born in 1916–35 in Petrogradsky district, Russia	To determine whether starvation during increased growth periods have long term health consequences.	*Independent variables:* - Age at siege; - Food shortage; - Questionnaire to assess: marital status, education, occupational class, smoking and alcohol consumption. *Outcome parameters (in adults):* - BMI; - Height; - Skinfold thickness; - LDL/HDL cholesterol; - Blood pressure; - CHD (according to Rose questionnaire); - Data on mortality.	Men exposed to famine in puberty had an increased mortality from ischemic heart disease (RR 1.39, 95% CI 1.07–1.79) and stroke (1.67, 1.15–2.43), including hemorrhagic stroke (1.71, 0.90–3.22).
Koupil, 2007 [25]	Five thousand six hundred and thirty-four subjects, resident in St. Petersburg between 1975–1982: 3905 men born between 1916–1935; 1729 women born between 1910–1940.	To investigate the long-term consequences of the food deprivation on cardiovascular risk factors and mortality in surviving adults.	*Independent variables:* - Age at siege; - Food shortage; - Questionnaire to assess: marital status, education, occupational class, smoking and alcohol consumption. *Outcome parameters (in adults):* - BMI; - Height; - Skinfold thickness; - LDL/HDL cholesterol; - Blood pressure; - CHD (according to Rose questionnaire); - Data on mortality.	Higher mean SBP among women who experienced the severest starvation at age 6–8 years and in men who were exposed to the starvation at age 9–15 years: age adjusted differences in SBP were 7.4 (95% CI: −1.4, 16.2) mm Hg in women and 3.3 (95% CI: 1.1, 5.5) mm Hg in men. Exposure to siege was associated with a statistically significant excess among men only, with a fully adjusted overall OR for men exposed at age 6–25 of 1.20 (95% CI: 1.03, 1.39) for mean blood pressure.
Rotar, 2015 [81]	Three hundred and six subjects of 64–81 years, who experienced famine during the Siege; 51 age and sex-matched subjects, aged 67–82, non-exposed.	To assess cardiovascular health, markers of cardiovascular aging and telomere length in survivors of the Siege of Leningrad.	*Independent variables:* - Age at siege; - Food shortage; - Questionnaire to assess: marital status, education, occupational class, smoking and alcohol consumption. *Outcome parameters (in adults):* - BMI; - Height; - Weight; - Skinfold thickness; - LDL/HDL cholesterol; - Glucose level; - Blood pressure; - CHD (according to Rose questionnaire); - Data on mortality; - Echocardiography; - ECG; - Blood samples for relative telomere length measurement by quantitative Polymerase Chain Reaction (PCR).	Both men and women exposed had shorter telomere length: T/S ratio 0.44 (0.25; 0.64) vs. controls 0.91 (0.47; 1.13) (*p*-value < 0.0001).
The Biafran Study Cohort (cohort study, Enugu, Nigeria)				
Hult, 2010 [27]	One thousand three hundred and thirty-nine adults, from the Human group igbo, born between 1965–1973, Nigeria: 388 exposed to famine in early childhood (born 1965–1967); 292 exposed to fetal-infant famine (born 1968–January 1970); 486 unexposed (born 1971–1973); 173 in transitional period (Februar–December 1970).	To study the risks for hypertension, diabetes and overweight in adults, after fetal and infant exposure to famine.	*Independent variables:* - Birth date in relation to Biafran Famine; - Sex, age, smoking status, education. *Outcome parameters (in adults):* - BMI; - Height; - Weight; - Waist circumference; - Glucose level; - Blood pressure.	OR and CI for 40 years-old subjects exposed to famine: - SBP ≥ 140: childhood (1.78 [1.19–2.68]), fetal-infant (3.02 [2.01–4.52]), unexposed (1); - DBP ≥ 90: childhood (1.35 [0.93–1.97]), fetal-infant (2.49 [1.72–3.62]), unexposed (1); - Severe HT: childhood (1.36 [0.63–2.93]), fetal-infant (2.68 [1.31–5.46]), unexposed (1); - IGT: childhood (1.15 [0.70–1.86]), fetal-infant (1.76 [1.09–2.85]), unexposed (1); - Diabetes: childhood (1.88 [0.66–5.33]), fetal-infant (3.11 [1.14–8.51]), unexposed (1); - Overweight: childhood (1.02 [0.77–1.34]), fetal-infant (1.41 [1.03–1.93]), unexposed (1); - Obesity: childhood (1.20 [0.87–1.67]), fetal-infant (1.30 [0.92–1.85]), unexposed (1).
